# Evaluation of Serum Amylase and Lipase in Gestational Diabetes Mellitus and Association With Gastrointestinal Symptoms

**DOI:** 10.7759/cureus.48376

**Published:** 2023-11-06

**Authors:** Aradhana Singh, Raj K Singh, Amresh Kumar Singh, Mudit Chauhan, Vibha Gautam

**Affiliations:** 1 Department of Obstetrics and Gynaecology, All India Institute of Medical Sciences, Gorakhpur, IND; 2 Department of Medicine, Baba Raghav Das Medical College, Gorakhpur, IND; 3 Department of Microbiology, Baba Raghav Das Medical College, Gorakhpur, IND; 4 Department of Community Medicine, Baba Raghav Das Medical College, Gorakhpur, IND; 5 Department of Obstetrics and Gynaecology, Baba Raghav Das Medical College, Gorakhpur, IND

**Keywords:** gestational diabetes mellitus (gdm), gi symptoms in pregnancy, lipase, amylase, exocrine pancreas

## Abstract

Introduction: Gestational diabetes mellitus (GDM) is a common complication during pregnancy, with potential adverse effects on maternal and fetal health. Several studies have reported that in diabetic patients, both morphological and functional pathological mechanisms lead to exocrine pancreatic dysfunction. Pancreatic enzyme deficiency or dysfunction result in the inability to digest food properly, giving rise to a range of gastrointestinal (GI) symptoms. We hypothesized that pregnant women with GDM may also have deficiency of pancreatic enzymes, amylase and lipase, leading to persistent GI symptoms beyond the first trimester and impaired quality of life.

Objective: The objective of this study was to evaluate serum amylase and lipase levels in pregnant women with GDM and association with GI symptoms. Understanding the relationship between GDM and exocrine pancreatic function may help identify novel therapeutic targets and improve the clinical management of GDM women with GI symptoms.

Materials and methods: This cross-sectional comparative study included a total of 125 pregnant women in their third trimester, who were either diagnosed with diabetes (n = 25) or were healthy volunteers without diabetes (n = 100). A detailed history, including the presence or absence of GI symptoms and the type of symptoms, was recorded. Serum amylase and lipase levels were measured using enzyme kinetic assay. Data were coded and analysed.

Results: GI symptoms were significantly more in GDM women than in normal pregnant women, and GDM women with GI symptoms had significantly lower levels of serum lipase and amylase than normal pregnant women with GI symptoms.

Conclusion: The study suggests the importance of evaluating serum amylase and lipase in GDM women with GI symptoms, as they may be indicative of underlying pancreatic enzyme deficiency.

## Introduction

Gestational diabetes mellitus (GDM) is a common complication during pregnancy, affecting up to 14% of pregnant women worldwide [1]. However, there is a wide variability in reported prevalence estimates for GDM in India, varying from 3.8% to up to 17.9% [2,3,4]. GDM is characterized by high blood sugar levels during pregnancy, which can cause a range of adverse maternal and fetal outcomes. The pathophysiology of GDM involves insulin resistance and impaired insulin secretion, which may affect the function of other organs, including the pancreas itself.

The pancreas plays a crucial role in glucose homeostasis, by secreting hormones and by secreting digestive enzymes, including amylase and lipase, to break down carbohydrates and fats.

Exocrine pancreatic insufficiency (EPI) is a clinical syndrome that results from a deficiency of digestive enzymes secreted by the pancreas. EPI has been traditionally associated with chronic pancreatitis, cystic fibrosis and other pancreatic diseases, but recent studies have also reported EPI in patients with diabetes mellitus (DM) [5,6].

The association between DM and EPI has been attributed to multiple factors, including chronic inflammation, oxidative stress and impaired islet-acinar interactions. In diabetic patients, hyperglycemia has been shown to stimulate the secretion of pro-inflammatory cytokines, leading to inflammation and fibrosis in the pancreas. This inflammatory response can damage the acinar cells responsible for producing digestive enzymes and cause EPI. In addition, oxidative stress, which is known to be elevated in diabetes, can further contribute to exocrine pancreatic injury and dysfunction [7].

Several studies have reported a correlation between EPI and GI symptoms in diabetic patients, including bloating, diarrhoea, flatulence and abdominal pain [8,9]. These symptoms can be attributed to the inability of the pancreas to produce and secrete digestive enzymes properly, leading to malabsorption of nutrients and GI discomfort. Moreover, previous studies have reported that patients with DM may have reduced serum amylase and lipase levels, which are indicators of pancreatic function [7,10,11]. However, the association between GI symptoms and serum amylase and lipase levels has not been evaluated in GDM previously.

Considering the above observations, we hypothesized that pregnant women with GDM may also have reduced serum amylase and lipase levels, leading to morbidity due to GI symptoms beyond the first trimester. Therefore, the objective of this study was to evaluate serum amylase and lipase levels in pregnant women with GDM and association with GI symptoms. Understanding the relationship between GDM and exocrine pancreatic function may help identify novel therapeutic targets to reduce morbidity and improve clinical management of GDM women with GI symptoms.

In the absence of direct evidence, it may be useful to consider the existing literature on the relationship between serum amylase and lipase levels and GI symptoms in other diabetic populations. Although there is some evidence to suggest that serum amylase and lipase levels may be reduced in diabetic patients, the extent to which this is associated with GI symptoms is not well established. It is possible that other factors, such as alterations in gut microbiota or dietary changes, may also play a role in the development of GI symptoms in GDM [12].

Given the high prevalence of GDM and the potential impact on maternal and fetal health, it is important to further investigate the relationship between serum amylase and lipase levels and GI symptoms in this population. Understanding the underlying mechanisms and potential therapeutic interventions could lead to improved management of GDM and better outcomes for both mothers and their babies.

## Materials and methods

The study was conducted at the obstetrics outpatient department (OPD) of Nehru Chikitsalay Baba Raghav Das Medical College (BRDMC), Gorakhpur, over a period of one year from November 2021 to December 2022. The study included pregnant women in their third trimester as its study population. This research followed a comparative cross-sectional study design.

There is a wide variability in reported GDM prevalence across different regions in India. To determine the sample size, the prevalence of GDM in pregnant females was estimated to be 6.6% (p), based on a pilot study we conducted to determine GDM prevalence in this region. The formula n = 4pq/l^2^ was employed, where q=(1-p) and absolute error (l) of 4.5 was considered. The calculation resulted in a sample size of 121.76, which was rounded off to 125 for the sake of convenience. Consecutive sampling was done, and 125 pregnant women in their third trimester, attending the obstetric OPD were enrolled, rigorously following the inclusion and exclusion criteria and consenting to participate. Out of the total 125 participants enrolled, 25 had GDM, and the remaining 100 had no GDM.

Institutional Human Ethics Committee of Baba Raghav Das Medical College, Gorakhpur, issued approval 5-A/IHEC/2021. Ethical committee approval was obtained before data collection for all participants, ensuring proper consent was acquired. Exclusions from the study was made for women who were critically ill and with conditions other than diabetes that can potentially affect serum amylase and lipase levels.

A detailed history regarding demographic and clinical profile was obtained for all participants. The presence or absence of gastrointestinal symptoms and the type of symptoms were also recorded. Clinical examinations and laboratory investigations were carried out on all women, including routine antenatal investigations, fasting blood sugar (FBS), post-prandial blood sugar (PPBS), liver function test (LFT) and kidney function test (KFT), using an automated analyser.

Serum amylase and lipase levels were measured using an enzyme kinetic assay. Hemoglobin A1C (HbA1C) was measured by high-performance liquid chromatography (D-10 Testing Program, Bio-Rad, USA). The study compared the findings between the GDM and non-GDM groups. 

Statistical analysis

Data were coded and analysed using IBM SPSS Statistics for Windows, version 23 trial version (released 2015; IBM Corp., Armonk, New York, United States). Descriptive statistics was elaborated in the form of means/standard deviation, continuous variable in the form of median/interquartile ranges (IQRs) and categorical variable in the form of frequencies and percentage. Group comparison for continuously distributed data were made using independent sample t-test, and for non - normally distributed data, the Wilcoxon test was used. Chi-squared data were used for group comparison in categorical data. In case the expected frequency in the contingency table was found to be <5 for >25% of the cells, Fisher’s exact test was used instead. Statistical significance was kept at p<0.05.

## Results

There was no significant difference between the groups in terms of the distribution of parity (W = 1535.500, p = 0.067) and period of gestation (POG) (weeks) (W = 979.500, p = 0.094). The mean (SD) parity in the GDM and non-GDM groups was 3.00 (1.78) and 2.24 (0.98), respectively. The mean SD of POG (weeks) in the GDM and non-GDM groups was 34.72 (4.36) and 35.73 (5.08), respectively.

However, there was a significant difference between the groups in terms of the distribution of weight (χ2 = 9.483, p = 0.041) and age (χ2 = 10.348, p = 0.006). The mean (SD) of weight (kg) in the GDM and non-GDM groups was 62.08 (8.56) and 57.98 (7.16), respectively. The mean (SD) of age (years) in the GDM and non-GDM groups was 30.44 (4.67) and 26.73 (3.95), respectively (Table 1).

**Table 1:** Association between the parameters and GDM (n = 25) and non-GDM groups (100) POG: period of gestation, GDM: gestational diabetes mellitus. **Significant at p<0.05, 1: Wilcoxon-Mann-Whitney U test, 2: Chi-squared Test, 3: Fisher's exact test, 4: t-test

Parameters	Group		p value
	GDM (n = 25)	Non-GDM (n = 100)	
Age (years)	30.44 ± 4.67	26.73 ± 3.95	<0.001^1^
Age**			0.006^2^
20-25 years	4 (16.0%)	45 (45.0%)	
26-35 years	14 (56.0%)	46 (46.0%)	
36-42 years	7 (28.0%)	9 (9.0%)	
Parity	3.00 ± 1.78	2.24 ± 0.98	0.067^1^
Parity category			0.428^2^
P1	4 (16.0%)	26 (26.0%)	
P2	8 (32.0%)	35 (35.0%)	
≥P3	13 (52.0%)	39 (39.0%)	
POG^⁎^ (weeks)	34.72 ± 4.36	35.73 ± 5.08	0.094^1^
POG			0.069^3^
<28 weeks	1 (4.0%)	5 (5.0%)	
28-36 weeks	16 (64.0%)	39 (39.0%)	
≥37 weeks	8 (32.0%)	56 (56.0%)	
Weight (kg)	62.08 ± 8.56	57.98 ± 7.16	0.034^4^
Weight**			0.041^3^
41-50 kg	3 (12.0%)	21 (21.0%)	
51-60 kg	9 (36.0%)	45 (45.0%)	
61-70 kg	9 (36.0%)	32 (32.0%)	
71-80 kg	4 (16.0%)	2 (2.0%)	

Chi-squared test was used to explore the association between 'group' and 'socioeconomic status'. There was no significant difference between the groups in terms of the distribution of socioeconomic status (χ2 = 5.654, p = 0.059) (Table 2).

**Table 2:** Association between group and socioeconomic status (n = 125) GDM: gestational diabetes mellitus

Socioeconomic status	Group			Chi-squared test	
	GDM (n = 25)	Non-GDM (n = 100)	Total	χ2	p-value
Lower middle	15 (60.0%)	41 (41.0%)	56 (44.8%)	5.654	0.059
Upper lower	10 (40.0%)	43 (43.0%)	53 (42.4%)		
Lower	0 (0.0%)	16 (16.0%)	16 (12.8%)		

Chi-squared test was used to explore the association between 'group' and 'GI symptoms'. There was a significant difference between the groups in terms of the presence of GI symptoms (χ2 = 18.883, p = <0.001). A portion (64.0%) of the participants in the GDM group had GI symptoms, while only 20.0% of the participants in the non-GDM group had GI symptoms (Table 3).

**Table 3:** Association between the group and GI symptoms (n = 125) GI: gastrointestinal; GDM: gestational diabetes mellitus

GI symptoms	Group			Chi-squared test	
	GDM (n = 25)	Non-GDM (n = 100)	Total (125)	χ2	p-value
Present	16 (64.0%)	20 (20.0%)	36 (28.8%)	18.883	<0.001
Absent	9 (36.0%)	80 (80.0%)	89 (71.2%)		

Fisher's exact test was used to explore the association between 'group' and 'GI symptom details'. There was no significant difference between the groups in terms of the distribution of GI symptom details (χ2 = 0.279, p = 1.000). The most common GI symptom in both groups was constipation (Table 4).

**Table 4:** Association between the group and GI symptom details (n = 36) GI: gastrointestinal; GDM: gestational diabetes mellitus

GI symptom details	Group			Fisher's exact test	
	GDM (n = 16)	Non-GDM (n = 20)	Total (36)	χ2	p-value
Constipation	6 (37.5%)	7 (35.0%)	13 (36.1%)	0.279	1.000
Abdominal pain	3 (18.8%)	5 (25.0%)	8 (22.2%)		
Vomiting	4 (25.0%)	4 (20.0%)	8 (22.2%)		
Bloating	3 (18.8%)	4 (20.0%)	7 (19.4%)		

The mean (SD) of serum lipase (IU/L) in the GDM group was 37.04 (16.52) and that in the non-GDM group was 46.80 (33.06). There was no significant difference between the groups in terms of serum lipase (IU/L) (W = 977.500, p = 0.093) (Table 5).

**Table 5:** Comparison of the groups in terms of serum lipase (IU/L) (n = 125) SD: standard deviation; GDM: gestational diabetes mellitus; IQR: interquartile range

Serum lipase (IU/L)	Group		Wilcoxon-Mann-Whitney U test	
	GDM (n = 25)	Non-GDM (n = 100)	W	p-value
Mean (SD)	37.04 (16.52)	46.80 (33.06)	977.500	0.093
Median (IQR)	32.6 (22.3-48.6)	41.1 (28.35-57.37)		
Range	18.3 - 85.6	18.1 - 310.8		

There was a significant difference between the groups in terms of serum amylase (IU/L) (W = 734.500, p = 0.001), with the median serum amylase (IU/L) being lowest in the GDM group (43.4 (35.9-61.3) vs. 62.4 (45.98-91.15)) (Table 6)

**Table 6:** Comparison of the groups in terms of serum amylase (IU/L) (n = 125) SD: standard deviation; GDM: gestational diabetes mellitus; IQR: interquartile range

Serum amylase (IU/L)	Group		Wilcoxon-Mann-Whitney U test	
	GDM (n = 25)	Non-GDM (n = 100)	W	p-value
Mean (SD)	48.88 (18.98)	71.92 (39.13)	734.500	0.001
Median (IQR)	43.4 (35.9-61.3)	62.4 (45.98-91.15)		
Range	22.2 - 86.4	21.2 - 270		

There was a significant difference between the groups with GI symptoms present (n = 36) in terms of serum lipase (IU/L) (W = 26.000, p = <0.001), with the median serum lipase (IU/L) being lowest in the GDM group (Table 7).

**Table 7:** Comparison of the groups in terms of serum lipase (IU/L) in (GI symptoms: present) (n = 36) SD: standard deviation; GDM: gestational diabetes mellitus; IQR: interquartile range; GI: gastrointestinal

Serum lipase (IU/L)	Group		Wilcoxon-Mann-Whitney U Test	
	GDM (n = 16)	Non-GDM (n = 20)	W	p-value
Mean (SD)	34.81 (12.40)	77.65 (57.84)	26.000	<0.001
Median (IQR)	31.9 (26.3-47.25)	65.6 (50.33-82.93)		
Range	18.3 - 55.5	41.6 - 310.8		

There was a significant difference between the groups with GI symptoms present (n = 36) in terms of serum amylase (IU/L) (W = 0.000, p = <0.001), with the median serum amylase (IU/L) being lowest in the GDM group (Table 8).

**Table 8:** Comparison of the groups in terms of serum amylase (IU/L) (GI symptoms: present) (n = 36) SD: standard deviation; GDM: gestational diabetes mellitus; IQR: interquartile range; GI: gastrointestinal

Serum amylase (IU/L)	Group		Wilcoxon-Mann-Whitney U test	
	GDM (n = 16)	Non-GDM (n = 20)	W	p-value
Mean (SD)	51.07 (17.69)	125.06 (46.50)	0.000	<0.001
Median (IQR)	49.9 (36.2-63.35)	99.85 (93.98-154.82)		
Range	24.6 - 79.8	86.9 - 270		

There was no significant difference between the groups with GI symptoms absent in terms of serum lipase (IU/L) (W = 345.000, p = 0.844) (Table 9).

**Table 9:** Comparison of the groups in terms of serum lipase (IU/L) (GI symptoms: absent) (n = 89) SD: standard deviation; GDM: gestational diabetes mellitus; IQR: interquartile range; GI: gastrointestinal

Serum lipase (IU/L)	Group		Wilcoxon-Mann-Whitney U test	
	GDM (n = 09)	Non-GDM (n = 80)	W	p-value
Mean (SD)	40.99 (22.42)	39.08 (16.24)	345.000	0.844
Median (IQR)	43.4 (22.3-48.6)	32.75 (27.17-46.62)		
Range	19.3 - 85.6	18.1 - 93.7		

The difference between the groups in terms of serum amylase was significant (W = 210.000, p = 0.042). The median serum amylase (IU/L) was lowest in the GDM group with GI symptoms absent (36.5 (35.7-40.3) vs. 53.2 (42.38-70.45) (Table 10).

**Table 10:** Comparison of the groups in terms of serum amylase (IU/L) (GI symptoms: absent) (n = 89) SD: standard deviation; GDM: gestational diabetes mellitus; IQR: interquartile range; GI: gastrointestinal

Serum amylase (IU/L)	Group		Wilcoxon-Mann-Whitney U test	
	GDM (n = 09)	Non-GDM (n = 80)	W	p-value
Mean (SD)	44.99 (21.61)	58.64 (22.48)	210.000	0.042
Median (IQR)	36.5 (35.7-40.3)	53.2 (42.38-70.45)		
Range	22.2 - 86.4	21.2 - 122.5		

## Discussion

Several literature works over the last two decades have shown that diabetes mellitus is associated with morphological and functional impairment of exocrine pancreas [13,14]. Pancreatic endocrine and exocrine cells develop from a common progenitor cell population. The physical proximity of endocrine cells to exocrine cells supports the idea that crosstalk may be possible between different pancreatic cell types [15].

GI symptoms in diabetes, such as abdominal pain, flatulence, bloating, vomiting, diarrhoea and constipation, may be related to the insufficient secretion of pancreatic enzymes, such as amylase, lipase and protease and/or sodium bicarbonate. These wide range of clinical symptoms markedly impair the quality of life in diabetics and might respond to enzymatic treatment [16].

Association between GI symptoms and level of exocrine pancreatic enzymes in GDM has not been studied previously. This study was done with the objective to find an association of GI symptoms with serum amylase and lipase level in GDM and normal pregnant women. 

There was no statistically significant difference in parity, period of gestation (weeks) (Table 1) and the socioeconomic status (Table 2) between the two groups. Most participants in both groups had POG between 28 and 36 weeks. The majority of the participants in both groups were between 26 and 35 years of age. However, the mean age (years) in the GDM group was significantly more than that in the non-GDM group (30.44 (4.67) vs. 26.73 (3.95) p=0.006). The mean weight was higher in the GDM group (62.08 ± 8.56) compared to the non-GDM group (57.98 ± 7.16), and the difference was statistically significant (p = 0.0344) (Table 1). The difference in weight and age between the two groups may be because of the association of GDM with pregnancy weight gain and advancing maternal age.

GI disorders represent some of the most frequent complaints during pregnancy. Till now, the reasons are usually attributed to elevated levels of progesterone (e.g., nausea/vomiting, constipation, gastroesophagel reflux disease (GERD)) and/or prostaglandins (diarrhoea) [12], but in GDM women, exocrine pancreatic dysfunction must be thought of and excluded, especially if GI symptoms persist in the second and third trimesters.

This study showed that a significantly higher proportion of pregnant women with diabetes had GI symptoms (64.0%) compared to non-diabetics (20.0%) (p-value < 0.001), indicating a strong association of GI symptoms with GDM (Table 3).

The GI symptoms reported in the GDM women included constipation (37.5%), pain abdomen (18.8%), vomiting (25.0%) and bloating (18.8%). However, there was no significant difference in the distribution of these symptoms between the GDM and normal pregnant women (p = 1.000) (Table 4).

In a population-based study on 15,000 adults, GI symptoms were found to be more prevalent in diabetics compared to controls (9.7% vs. 4.6%) and was not associated with the duration of diabetes or type of diabetic treatment [17].

In another population-based cohort study of 110 young adults with long-standing diabetes, the author found increased prevalence of upper GI symptoms, such as anorexia and vomiting, among diabetics [18].

In terms of serum lipase levels, the mean and median levels were lower in the GDM women (37.04 ± 16.52 and 32.60 IU/L, respectively) compared to the normal pregnant women (46.80 ± 33.06 and 41.10 IU/L, respectively). However, the difference was not statistically significant (p = 0.0931) (Table 5).

Meanwhile, the mean and median serum amylase levels were significantly lower (p =0.001) in the GDM women (48.88 ± 18.98 and 43.40 IU/L, respectively) compared to the normal pregnant women (71.92 ± 39.13 and 62.40 IU/L, respectively), irrespective of the presence or absence of GI symptoms (Table 6).

In a case-control study of 90 subjects divided into three groups, including 30 apparently healthy controls, 30 cases of type 1 DM and 30 cases of type 2 DM, the authors found statistically significant (p < 0.01) low values for serum amylase and serum lipase in patients with type 1 and type 2 DM as compared to healthy controls. They suggested that serum amylase and serum lipase can be used as biochemical markers for the assessment of the pancreatic exocrine function [19].

In an epidemiological study, it was found that low serum amylase was associated with an increased risk of metabolic abnormalities, metabolic syndrome and diabetes. These results suggested a pancreatic exocrine-endocrine relationship [20]. Several other studies have quoted similar results [21].

In a single study evaluating serum amylase in GDM women, a total of 108 subjects fulfilled the diagnostic criteria of GDM, and the findings suggested that a low serum amylase level was significantly associated with increased risk of GDM [22]. However, no study was found exploring exocrine pancreas function and correlation with GI symptoms in GDM women.

The results from Tables 7 and Table 8 suggest that there is a strong association between GI symptoms and reduced exocrine pancreatic function. Serum lipase and serum amylase levels were significantly low among GDM women with GI symptoms in comparison with normal pregnant women with GI symptoms, as evidenced by the lower mean and median values in the GDM group compared to the normal pregnancy group. The levels of serum lipase and serum amylase are markers of pancreatic function, and their lower levels in the GDM women suggest that there may be some dysfunction in the exocrine pancreas of GDM women, which may be related to their GI symptoms. This finding is consistent with previous research that has shown that diabetic patients with GI symptoms often have an exocrine pancreatic dysfunction [23].

Serum lipase was not significantly different among both the GDM and non-GDM groups without GI symptoms (Table 9). However, serum amylase was significantly low in GDM women without GI symptoms (Table 10). This study showed that serum amylase was significantly lower in GDM women, while serum lipase was significantly low in GDM women with GI symptoms.

This may have important clinical implications, as the measurement of serum lipase and serum amylase levels can aid in the diagnosis and management of GDM women with GI symptoms. Further studies with larger sample sizes and robust statistical methods are needed to confirm the diagnostic utility of serum lipase and serum amylase in predicting GI symptoms in GDM women and to determine the clinical implications of these findings.

Limitations

It is important to note that the sample size in this study was relatively small, and the findings may not be generalizable to the larger population. The groups were also not comparable is all aspects. In addition, the study design was cross-sectional, and therefore, it cannot establish causality between GI symptoms and pancreatic dysfunction.

Further research with larger sample sizes and longitudinal study designs is needed to confirm these findings and explore the clinical implications of these results.

## Conclusions

The study found that GI symptoms, beyond the first trimester, were significantly more common among GDM women, compared to normal pregnant women. GDM women with GI symptoms exhibited lower serum amylase and lipase levels. Thus, the study provides valuable insights into the potential association between GDM and exocrine pancreatic enzyme deficiency in women presenting with GI symptoms. 

It is advisable to consider evaluating serum amylase and lipase level in GDM women experiencing GI symptoms, as these markers may serve as indicators of underlying exocrine pancreatic dysfunction in these specific subgroups of women. However, additional research is required to validate these findings and to gain a deeper understanding of the precise mechanism through which GDM may impact the exocrine pancreatic function.
